# Fortuitous discovery of an early neuroendocrine tumor during appendicular peritonitis

**DOI:** 10.1016/j.amsu.2022.104735

**Published:** 2022-09-22

**Authors:** Abdelilah El Bakouri, anas El wassi, Yassine Eddaoudi, Mounir Bouali, Khalid EL Hattabi, Fatimazahra Bensardi, Abdelaziz Fadil

**Affiliations:** a Visceral Surgery Emergency Department P35, University Hospital Center Ibn Rochd, Casablanca, Morocco; b Faculty of Medicine and Pharmacy, Hassan II University, Casablanca, Morocco

**Keywords:** Small bowel, Surgery, Neuroendocrine tumor, Carcinoide syndrome

## Abstract

**Introduction:**

Neuroendocrine tumors of the small bowel are rare but represent the most frequent histological type at this level; their incidence is increasing thanks to the evolution of diagnostic means.Intestinal NETs, developed at the expense of enterochromaffin cells of the embryological midgut, are frequently associated with mesenteric lymph node dissemination and distant metastasis (liver, peritoneum);

**Materials and methods:**

We report a case of Incidental discovery of a small bowel neuroendocrinetumor during appendicular peritonitis in the department of Emergency visceral surgery P35 of the ibn rochd hospital in casablanca.

**Results:**

Our patient Our patient was admitted to the emergency room for generalized abdominal pain with an appendicular symptomatology evolving five days before days the consultation with clinical examination: conscious patient stable on the hemodynamic and respiratory plan The examination noted generalized abdominal defense the hernial orifices were free The biological work-up revealed a hb 13 g/dL; hyperleukocytosis with predominantly PNN at 18,300 elements/mm3,CRP was elevated to 190, renal function was normal urea 5 mmol/L creatinemia 9 mg/l an abdominal ultrasound showed a 9 mm appenndix perforated at its tip with moderate peritoneal effusion.

the patient were operated in the emergency room, approached by laparotomy with the exploration we found a swollen and inflamed appenndix perforated at the level of its tip with moderate peritoneal effusion with false membranes in all the peritoneal cavity with the presence of a polyp localized at 2 m from the duodenojejunal flexur .

the patient benefited from a retrograde appendectomy with peritoneal cleansing and a resection of the polyp with 1cm on each side with a Grele-grele anastomosis with the examination of the anapathomopathologist: aspect compatible with a well differentiated neuroendocrine tumor of grade 2.

**Conclusion:**

Digestive NETs are rare tumors, but their incidence has increased significantly in recent years. This is due to a better knowledge of these tumors, whose diagnosis is becoming easier with the advent of new morphological and biological techniques.

The intestinal location is the most frequent. The digestive surgeon must therefore be familiar with its management. An update of knowledge and collaboration between surgeons, anatomopathologists, radiologists and oncologists are necessary, Whatever their location, these tumors are on the one hand capable of producing and secreting amines and on the other hand they are characterized by a common phenotype, expression of general endocrine markers (specific neuron enolase, chromogranin) or specific endocrine markers and expression of peptide receptors such as somastotatin receptors.These tumors are most often diagnosed incidentally during the workup of aspecific digestive disorders or during hormonal hypersecretion syndrome or rarely by a complication.

## Highlights

• Neuroendocrine tumors of the small bowel are rare but represent the most frequent histological type at this level.
• Their incidence is increasing thanks to the evolution of diagnostic means.
• These tumors are most often diagnosed incidentally during the workup of aspecific digestive disorders or during hormonal hypersecretion syndrome or rarely by a complication.

## 1 Introduction

Neuroendocrine tumors (NETs) are a group of tumors with the ability to secrete peptides or hormones capable of growing in any part of the body including the digestive tract, they are tumors corresponding to neoplastic proliferation from cells of the diffuse neuroendocrine system dispersed in the digestive tract .

The most frequent digestive NETs are those originating in the small intestine. They are characterized by a slow evolution. The clinical presentation is rarely immediately suggestive and the diagnosis is often made only at advanced stages.

Intestinal NETs, developed at the expense of enterochromaffin cells of the embryological midgut, are frequently associated with mesenteric lymph node dissemination and distant metastasis (liver, peritoneum); however, even in the case of associated metastases, their progression is often slow with a relatively long survival compared to NETs originating from the fore- and hindgut.

Whatever their location, these tumors are on the one hand capable of producing and secreting amines and on the other hand they are characterized by a common phenotype, expression of general endocrine markers (specific neuron enolase, chromogranin) or specific, and expression of peptide receptors such as somastotatin receptors.

These tumors are most often diagnosed incidentally during the workup of aspecific digestive disorders or during hormonal hypersecretion syndrome or rarely by a complication.

This case report is compliant with the SCARE Guidelines 2020 [29].

## 2 Patient and observation

**Fig. 1:**
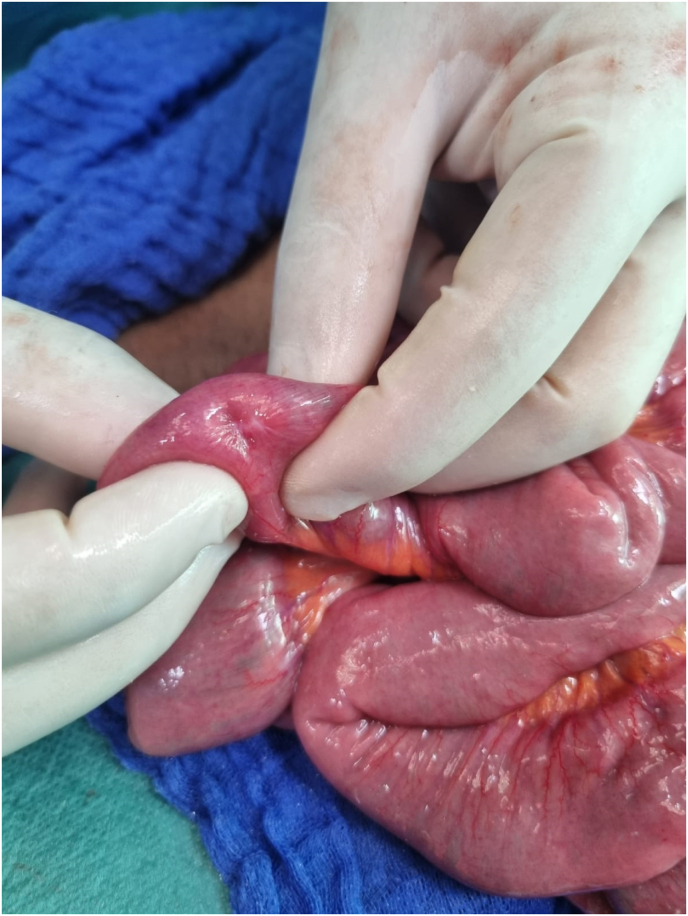
Intraoperative view showing.

A 38-year-old man chronic smoker was admitted to the emergency room for generalized abdominal pain with an appendicular origin with a symptomatology evolving five days before days before the consultation with clinical examination: noted generalized abdominal defense the hernial orifices were free The biological work-up revealed a hb 13 g/dL; hyperleukocytosis with predominantly PNN at 18,300 elements/mm3,CRP was elevated to 190, renal function was normal urea 5 mmol/L creatinemia 9 mg/l an abdominal ultrasound showed a 9 mm appenndix perforated at its tip with moderate peritoneal effusion the patients were operated on in the emergency department, approached by laparotomy on exploration we found a swollen and inflamed appendix perforated at its tip with moderate peritoneal effusion with false membranes throughout the peritoneal cavity with the presence of a polyp 1.5 cm in diameter located 2 m from the duodenojejunal flexur and away from the appendix and the cecum 1 m away (Fig. 1).

The location of the polyp 1 m away from the cecum and appendix allowed us to avoid performing a right hemi colectomy and to adopt the following approach the patient benefited from a retrograde appendectomy with peritoneal cleansing and a resection of the polyp with 1cm on each side with a Grele-grele anastomosis with the examination of the anapathomopathologist: aspect compatible with a well differentiated neuroendocrine tumor of grade 2.

The patient has never had any symptoms that point to a carcinoid syndrome the surgical procedure was performed on a scheduled date with a correct pre-anesthetic assessment; the procedure was performed by an assistant professor in general surgery and two residents in the same specialty.

The operation was performed in the operating room of the P35 visceral emergency department at the CHU ibn rochd hospital in Casablanca, Morocco the patient was satisfied with the intervention and the improvement of his health in the short and long term.

## 3 Discussion

Neuroendocrine tumors of the small intestine, frequently called carcinoids, represent the majority of tumors of the small intestine. They develop on the serotonin-producing enterochromaffin cells. These tumors are characterized by a slow evolution with unspecific clinical signs, hence the diagnosis at advanced stages. Moreover, they are characterized by their high capacity of dissemination to the mesenteric lymph nodes. Due to the lack of specificity of the clinical signs, these tumors may be revealed only at the stage of complication, in particular by an ischemia of the intestine.

Primary malignant tumors of the small intestine are generally rare tumors. They represent 1–5% of all tumors of the digestive tract although the small intestine accounts for 75% of the total length and over 90% of the mucosal surface of the digestive tract [1]. Moreover, their incidence is particularly low due to the rapid regeneration of the mucosa of the small intestine, the low bacterial density that produce carcinogenic metabolites, the rapidity of transit that reduces the contact time of some carcinogens [2,3].

NETs are more frequently developed in the midgut (40%) where they are located in more than 85% of cases in the ileum [4].

Small bowel neuroendocrine tumors are a globally rare entity with a reported incidence of approximately 0.32/100,000 in the United Kingdom, 0.33/100,000 in Japan, 0.67/100,000 in the United States, 0.81/100,000 in Norway and 1.12/100,000 in Sweden according to the most recent data in 2016 [5].

In France, NETs represent the most frequent tumors of the small intestine with an incidence close to 1/100,000 inhabitants. They would represent between 30 and 50% of small bowel tumors, their prevalence being even higher on autopsy parts, reflecting the frequency of asymptomatic forms [6].

Several international studies show an increase in the incidence of intestinal NETs during the last decades. Better knowledge and improved detection techniques are responsible for this increase.

Neuroendocrine tumors of the intestine originating from enterochromaffin cells are sporadic, no genetic predisposition has been found; tumorigenesis is therefore secondary to acquired mutations in the genome of the neoplastic cells [11]: chromosomal instability, copy number aberrations, point mutations, methylation abnormalities, and suppression of inhibitory pathways [3,7].

Small bowel carcinoids therefore occur mainly in the ileum (more than 50%) and distal jejunum [8].

Appendicular neuroendocrine tumors are rare in adults and account for 3% of total NETs [9], this may be explained by the rarity of metastases implying that these tumors are less reported than those occurring in other sites. Duodenal NETs are rare, less than 2% of all gastrointestinal NETs. The different types of duodenal NETs are: gastrinomas 48%–66% of duodenal NETs, somatostatinomas 15%–43%, non-functional NETs 19%–27% [10,16].

The average age of diagnosis of these tumors is between 60 and 66 years [6,9,14], the appendicular localization is the most frequent in children [12,13].

NETs can be revealed by Clinical symptoms related to a hormonal hypersecretion syndrome for functional tumors, such as carcinoid syndrome or Atypical tumor syndrome for advanced stages or Digestive symptoms such as intermittent abdominal pain or the occurrence of Koenig syndrome. Abdominal pain is the most frequent sign [5]. A complication such as digestive hemorrhage, intestinal occlusion, entero-mesenteric ischemia, etc. may be present.

The discovery may be fortuitous during an endoscopic or radiological examination or a surgical exploration carried out in the context of aspecific digestive symptoms or during the histological examination of an operative specimen.

The main markers are chromogranin A (CgA), NSE and 5HIAA. The European Neuroendocrine Tumor Society (ENETS) recommends the systematic determination of CgA and urinary 5-HIAA for gastrointestinal NETs, for jejuno-ileal NETs these are the two systematic examinations to be performed in the first line [5].

Cross-sectional imaging methods (CT and MRI) are central examinations for the initial diagnosis and extension assessment. They allow knowledge of the tumor site and look for vascular or biliary invasion [15].

Intestinal NETs are typically hypervascular. They enhance after injection of iodinated contrast medium at the early (about 30 seconds after injection) and late (30 seconds) arterial phases, and wash out during the portal phase (70 seconds).

Thus, it is recommended to perform a thoracoabdomino-pelvic CT with acquisitions at the late arterial time (30 seconds) and then at the portal venous time (70–90 seconds), because some highly vascularized NETs are only visible at one or the other of these two phases (The first acquisition at the arterial time should concern the abdomen and the portal phase should concern the thorax, abdomen and pelvis) [17].

MRI with gadolinium injection and diffusion sequences is more sensitive than CT for the detection of liver and bone metastases [18,20]. Abdominopelvic MRI is recommended in combination with thoracic-abdominopelvic CT in order to perform an exhaustive search for metastases. The most efficient sequences are the hepatic arterial phase after injection of gadolinium and the T2 fast spin-echo sequence, but especially the diffusion sequences.

MRI remains the best examination for the detection of liver metastases with a very good sensitivity, which makes it the examination of choice for the detection and follow-up of liver metastases [19].

The therapeutic management must meet certain requirements: the treatment must be curative as far as possible. The objective must always be to maintain a good quality of life for as long as possible. The management must allow: Symptomatic treatment to control the manifestations of the carcinoid syndrome and to limit tumor secretion; curative excision of the tumor, lymphatics and satellite metastases or, failing that, to reduce the tumor volume as much as possible (maximum tumor cytoreduction) and to limit its extension; and finally, avoidance of complications and treatment [5,[24], [25], [26], [27], [28]].

Nutritional status should be systematically assessed and malnutrition treated.

It may be necessary to supplement common deficiencies in vitamins B3 (niacin), B12 and fat-soluble vitamins.

Medical treatment is essentially an anti-secretory treatment. It is a priority and must be started as soon as the blood sample is taken to measure the plasma markers.

Surgical removal is the only curative treatment for gastrointestinal NETs. Its purpose is three fold [21]: firstly, curative, as surgery is the only curative treatment.secondly, to allow control of an endocrine secretory syndrome if it is not sufficiently controlled by medical treatment alone or to potentiate the effect of medical treatment by reducing the tumor mass.and finally the prevention and management of local complications.

There are two types of surgery for GI NETs: "Curative" removal. It applies to the primary tumor and its possible lymph node and hepatic metastases. Cytoreductive surgery is rarely indicated if the hormonal syndrome is poorly controlled, provided that at least 80% of the tumor mass can be resected.

The procedure must be adapted to the location, size and extension of the tumour.

For G1 and G2 well-differentiated small bowel tumors, the reference treatment is surgical resection of the primary tumor: resection of a small bowel loop and its mesentery is necessary while respecting the superior mesenteric axis as well as associated mesenteric lymph nodes, with the objective of sparing as much small bowel length as possible. This surgery reduces the risk of subsequent local complications (mainly occlusion, which can occur in 20–30% of cases).

There are few data in the literature concerning the treatment of well-differentiated G3 NETs. Surgery seems to be the first therapeutic option for these tumors. The recommendations for surgical treatment are similar to those for "aggressive" G2 tumors [22,23].

Prognostic evaluation of NETs of the gastrointestinal tract is based on their histopronostic classification: the degree of differentiation and the proliferation index, which are major independent prognostic factors. It has been shown that the Ki67 proliferation index is inversely correlated with survival. Labeling above 2% of cells indicates tumor aggressiveness.

## 4 Conclusion

Digestive NETs are rare tumors, but their incidence has increased significantly in recent years. This is due to a better knowledge of these tumors, whose diagnosis is becoming easier with the advent of new morphological and biological techniques.

The intestinal location is the most frequent. The digestive surgeon must therefore be familiar with its management. An update of knowledge and collaboration between surgeons, anatomopathologists, radiologists and oncologists are necessary.

Anatomopathology remains essential and indispensable for the diagnosis of NETs. It allows to confirm the diagnosis of NET and to distinguish well-differentiated tumors from poorly differentiated NECs which are two very different entities both therapeutically and evolutionarily.

The prognosis is essentially determined by the histological grade and the TNM stage.

Management is often multidisciplinary and therapeutic decisions must be made in multidisciplinary consultation meetings.

Surgery, the only curative treatment for localized endocrine tumors, plays a major role in the therapeutic strategy.

Control of clinical symptoms related to hormonal hypersecretion is imperative: it not only improves quality of life but also prevents the dreaded complications of carcinoid syndrome.

Surgery can meet two objectives, to treat the tumor syndrome and the secretory syndrome, with the lowest possible morbidity, taking into account the slow evolution and the long survival observed.

Surveillance should be performed for several years under the responsibility of the surgeon. The modalities of surveillance depend on the type of resection, the tumor stage and the presence of metastases.

The prognosis is mainly determined by the histological grade and the TNM stage.

## Provenance and peer review

Not commissioned, externally peer-reviewed.

## Ethical approval

I declare on my honor that the ethical approval has been exempted by my establishment.

## Sources of funding

None.

## Author contribution

El wassi Anas: writing the paper and operating surgeon.

Eddaoudi Yassine: Corresponding author writing the paper and operating surgeon.

El Bakouri Abdelilah: writing the paper and operating surgeon.

Bouali Mounir: study concept.

El Hattabi Khalid: study concept.

Bensardi Fatimazahra: study concept.

Fadil Abdelaziz: correction of the paper.

## Registration of research studies

Researchregistry2464.

## Guarantor

Dr ED ELW.

## Consent

Written informed consent for publication of their clinical details and/or clinical images was obtained from the patient.

## Declaration of competing interest

The authors declare having no conflicts of interest for this article.
